# Supplementary material for: High-throughput PBK modelling for dermal exposure: a pragmatic approach to predict systemic pharmacokinetics
Source: Arch Toxicol. 2026 Mar 31;100(6):2421–32. doi: 10.1007/s00204-026-04357-4 (PMC13221391; doi:10.1007/s00204-026-04357-4)
Supplement: Supplementary file 1 — Supplementary file1 (DOCX 670 kb) [file 204_2026_4357_MOESM1_ESM.docx]

# Supplementary Material

| 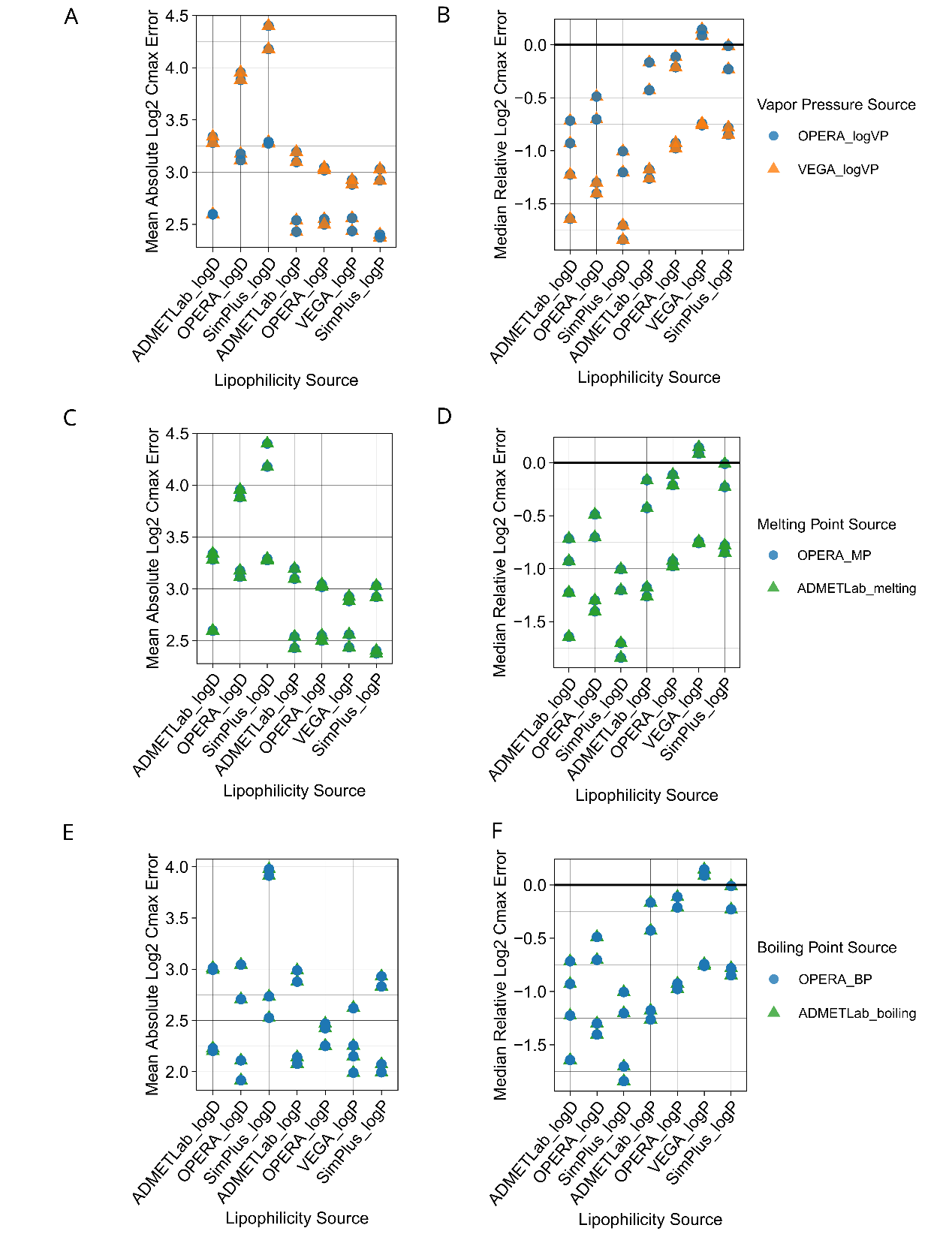 |
| --- |
| **Supplementary Fig. 1 Comparison of systemic PK prediction performances for different vapour pressure, melting and boiling point prediction sources.** Dermal absorption simulations of all parameterisation sources were examined against the collected *in vivo* dermal PK dataset. Solubility values were taken from SimPlus LogS. The first columns show Mean Absolute Log2 Cmax Errors (A, C, E) for different lipophilicity prediction sources. The second column shows Median Relative Log2 Cmax Errors (B, D, F)**.** VP stands for vapour pressure, MP is melting point, BP is boiling point. |

| 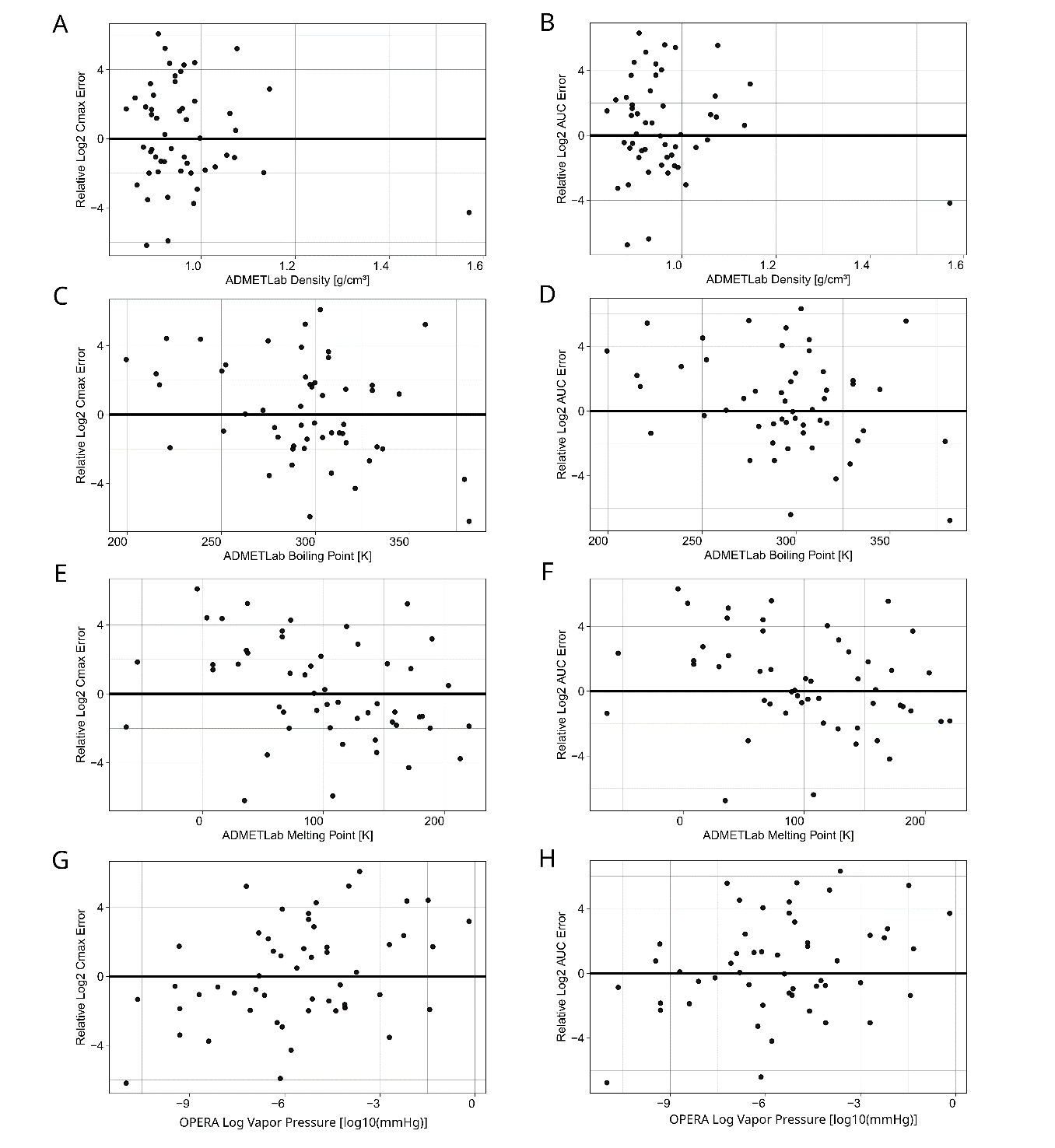 |
| --- |
| **Supplementary Fig. 2 Correlation of PK prediction errors of the best dermal HT-PBK strategy with additional compound properties.** The predicted compound parameters are compared against the Relative Log2 Cmax and AUC errors. |

| 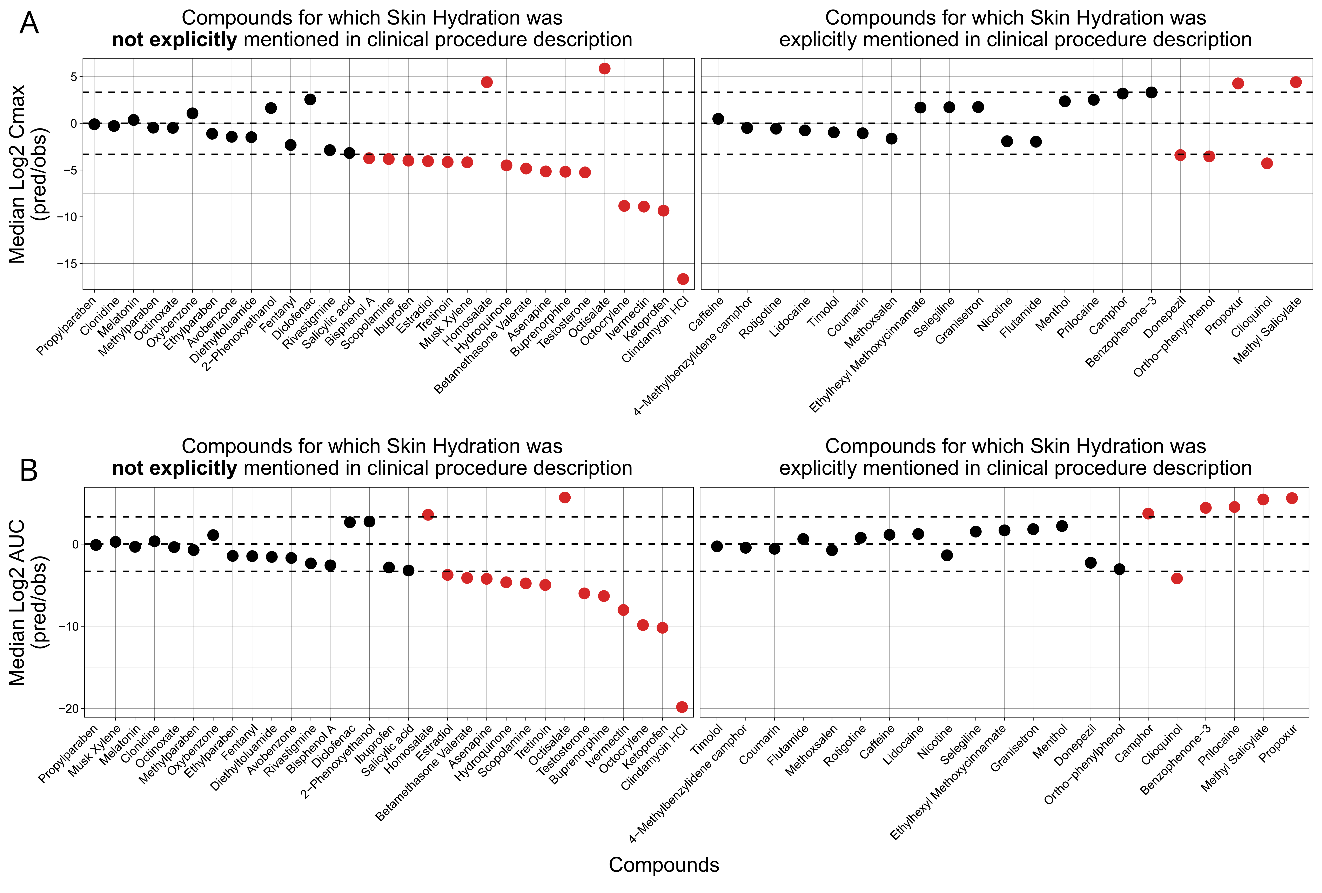 |
| --- |
| **Supplementary Fig. 3 The effect of unspecified skin hydration status on systemic PK predictions.** Many dermal PK studies did not explicitly mention whether application was performed on hydrated or non-hydrated skin. Shown results are for the assumption that lack of explicit mentioning relates to absence of skin hydration in study procedures (A) The Median Log2 Cmax predicted/observed values of each compound in the dataset. (B) The Median Log2 AUC predicted/observed values of each compound in the dataset. Plots are also split according to which information about the hydration status of the stratum corneum were provided in the clinical study information. |

| 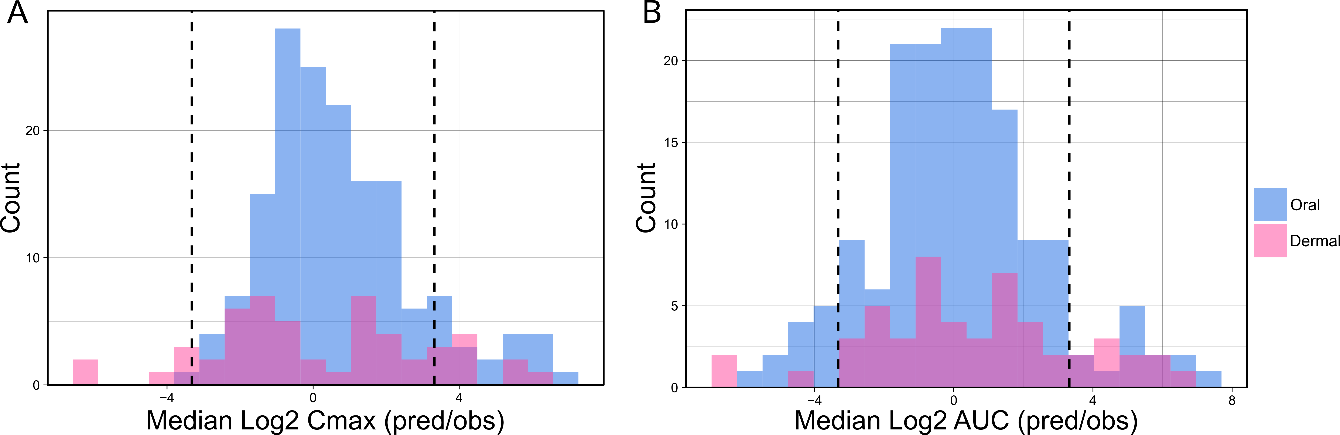 |
| --- |
| **Supplementary Fig. 4** **Comparison of non-normalised Cmax and AUC error distributions for dermal and oral PK predictions.** (A) The Median Log2 predicted/observed Cmax values of dermally administered compounds (pink) were compared against orally administered compounds (blue). (B) The Median Log2 predicted/observed AUC values of dermally administered compounds (pink) were compared against orally administered compounds (blue). Oral HT-PBK prediction errors were taken from Geci et al. 2024. |

| 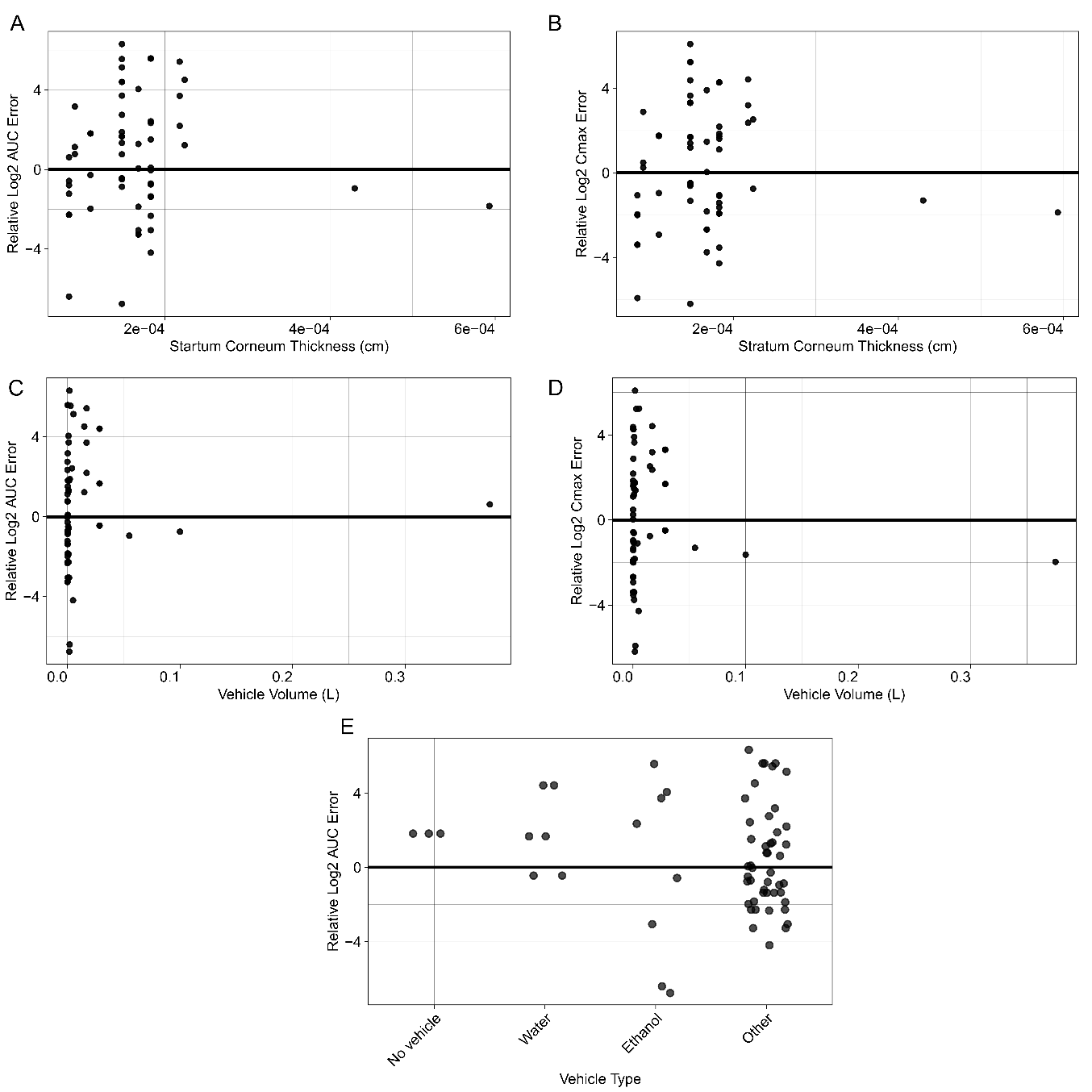 |
| --- |
| **Supplementary Fig. 5 Correlation of PK prediction errors of the best dermal HT-PBK strategy with application and skin physiology parameters.** Assumed stratum corneum thickness (AB), vehicle volume (CD) and vehicle type (E) are compared against the Relative Log2 Cmax and AUC errors. |
